# The Impact of Previous Pregnancy Loss on Lactating Behaviors and Use of Herbal Medicines during Breastfeeding: A Post Hoc Analysis of the Herbal Supplements in Breastfeeding InvesTigation (HaBIT)

**DOI:** 10.1155/2018/1035875

**Published:** 2018-11-08

**Authors:** Alessandra Bettiol, Niccolò Lombardi, Ettore Marconi, Giada Crescioli, Roberto Bonaiuti, Valentina Maggini, Eugenia Gallo, Alessandro Mugelli, Fabio Firenzuoli, Claudia Ravaldi, Alfredo Vannacci

**Affiliations:** ^1^Department of Neurosciences, Psychology, Drug Research and Child Health, Section of Pharmacology and Toxicology, University of Florence, Tuscan Regional Centre of Pharmacovigilance and Phytovigilance, Florence, Italy; ^2^Department of Experimental and Clinical Medicine, University of Florence, Florence, Italy; ^3^Center for Integrative Medicine, Careggi University Hospital, University of Florence, Florence, Italy; ^4^CiaoLapo Onlus, Charity for Healthy Pregnancy, Stillbirth and Perinatal Loss Support, Prato, Italy

## Abstract

**Introduction:**

Complementary and alternative medicines (CAMs) are commonly used among lactating women, despite the poor knowledge of these products and of their safety. Perception of pregnancy- and breastfeeding-related difficulties and consequent use of CAMs may differ in bereaved women, by force of the distress related to previous loss, although no literature evidence is available. This Herbal supplements in Breastfeeding InvesTigation (HaBIT) post hoc analysis explored the impact of previous pregnancy loss on lactating behaviors and on use of CAMs during breastfeeding.

**Methods:**

A web-based survey was conducted among lactating women with no previous alive child, resident in Tuscany (Italy). Data on lactating behavior and on CAMs use were collected and evaluated among women with previous pregnancy loss as compared to control women.

**Results:**

Out of 476 women answering the questionnaire, 233 lactating women with one child were considered. Of them, 80 had history of pregnancy loss. Cesarean birth was significantly more frequent among women with history of pregnancy loss as compared to controls (41% versus 22%;* p=0.004*). Proportion, length of exclusive breastfeeding, and occurrence of breastfeeding-related complications were comparable among the two cohorts. More than half of women used CAMs during breastfeeding. Use of CAMs was more frequent among women with previous pregnancy loss (54% versus 68%;* p=0.050)*, specifically considering herbal preparations (16% versus 30%;* p=0.018*). Major advisors for CAMs use were midwives. 18% and 23% of women without and with history of pregnancy loss declared no clear perception on CAMs efficacy and safety.

**Conclusion:**

Overcoming the social taboo of pregnancy loss and training healthcare professionals for an adequate management of the perinatal period are essential for an effective and safe care. Despite the common use and advice on CAMs use during breastfeeding, it is important to acknowledge that limited evidence supports their safety and efficacy during such critical period.

## 1. Introduction

Miscarriage and stillbirth are among the most impactful events in women's life. Miscarriage, defined as spontaneous loss of pregnancy within 24 weeks from conception, occurs in up to 15% of pregnancies [1]. Rate of stillbirth, i.e., spontaneous loss after 24 weeks of gestation, in high income countries is estimated to be 3.5 per 1000 total births [2].

The psychological implications of these events include depression, anxiety, grief, posttraumatic stress disorder [2, 3], and may impacts on the couple and family relationship, as well as on subsequent pregnancies [4, 5]. It is estimated that about 50% of couples that experience perinatal loss try to have a new pregnancy within a year [6, 7]. An integrative review of 15 articles points out that perinatal loss can overshadow the enjoyment and cause psychological distress in subsequent pregnancy [8], with evidence suggesting that anxiety, depression, and stress during pregnancy negatively impact on fetal and child development [9]. In addition, previous stillbirth is associated with increased risk of recurrent stillbirths, as well as with various adverse pregnancy outcomes, some of which are iatrogenic [10].

There is currently little evidence to guide healthcare providers in managing pregnancies subsequent to stillbirth [11, 12]. According to an Australian Internet-based survey, women often wish increased fetal surveillance and early delivery [13]. A web-based survey on 2716 parents, from 40 high- and middle-income countries, showed that pregnancies subsequent to stillbirth effectively had extra antenatal visits and ultrasound scan monitoring, but parents rarely had extra psychological support. Even when provided, support by healthcare providers was of poor quality, particularly considering listening and involvement in decisions [10]. Reasons for these unmet needs partially rely on the insufficient practice and training of healthcare providers caring for women experiencing a stillbirth: based on a nationwide cross-sectional survey, Italian healthcare providers felt inadequate and wanted professional training courses to better support bereaved families [14]. Poor support by trained healthcare personnel is not limited to the gestational period, but further involves the postpartum: lack of proper sanitary support in this phase is a major clinical issue, considering that breastfeeding may be related to different complications that may also require medications use. In a national web-based survey conducted on 388 lactating women in the frame of the Herbal supplements in Breastfeeding InvesTigation (HaBIT), 204 women declared use of complementary and alternative medicines (CAMs) during breastfeeding, mainly for the treatment of engorgement and breast fissures [15]. CAMs use was particularly high for women at the first child, and most of them informed their doctor of CAMs use during lactation. In fact, these kinds of treatments, being natural, are considered by women and some healthcare providers as safer compared to conventional drugs [16, 17]. The perception of an inadequate healthcare support may potentiate the tendency of women towards self-medication. This issue is of particular medical relevance, considering that CAMs' active ingredients are chemicals that share with traditional medications the potential to cause serious adverse effects [18]. To our knowledge, there is no evidence on attitudes towards the use of herbal medicines among breastfeeding women with previous pregnancy loss. Thus, the purpose of this* post hoc* analysis of the HaBIT study was to explore the impact of previous pregnancy loss on the lactating behaviors and on use of herbal medicines during breastfeeding.

## 2. Materials and Methods

### 2.1. Study Design

This is an observational study based on a web survey conducted over a six-year period (from February 1, 2012, to October 31, 2017). The methodology for data collection and the administered questionnaire have been extensively described elsewhere [15].

### 2.2. Data Collection

Briefly, a semistructured web-based questionnaire was administered using SurveyMonkey© web platform to a sample of lactating women, resident in Italy and attending the services of CiaoLapo Onlus, Charity for Healthy Pregnancy and Perinatal Loss Support. The 36-item questionnaire was designed and planned according to specific methodological literature [19–21] and was validated by an* ad hoc* panel of experts (pharmacologists, epidemiologists, toxicologists, pharmacists, and gynecologists) of the Tuscan Regional Centre of Pharmacovigilance and Phytovigilance and a clinician of the Center for Integrative Medicine.

The questionnaire provided information on (i) sociodemographic characteristics, including educational level, number of children, number of pregnancies, and type of delivery; (ii) lactating behavior and use of CAMs during current breastfeeding or during pregnancy; (iii) attitude towards CAMs in terms of perceived efficacy and safety; and (iv) benefits and adverse events experienced during CAMs use.

As for CAMs use during breastfeeding, reported products were classified in the following categories by a trained specialist by means of the European Pharmacopeia [22]: acupuncture, chiropractic/osteopathy/manual medicine, dietary supplements, domestic and traditional preparations, herbal preparations, homeopathy, natural galenical preparations, and phytotherapy.

### 2.3. Study Population

Based on provided answers, we included in the study only women that declared themselves to be lactating at the time of questionnaire or to have been lactating in the previous six months. We further excluded all women that declared themselves to have more than one child, thus limiting the study to the first breastfeeding. Based on the declared history of pregnancies, we stratified women into two classes: (i) women who reported no history of pregnancy loss (defined as “control women”) and (ii) women who declared one or more previous events of pregnancy loss.

### 2.4. Data Analysis

Sociodemographic characteristics, lactating behavior, use and attitude towards CAMs, and phytovigilance information were compared among women with versus without history of pregnancy loss.

Categorical variables were expressed as percentage value and compared using the Fisher exact test, while continuous variables were reported as median value and interquartile range (IQR) and compared using the Mann-Whitney test. A* p-value* <0.05 was considered statistically significant. All analyses were performed using the software STATA version 14.

## 3. Results

A total of 476 women responded to the web-based interview. After exclusion of nonlactating women (n=32), of questionnaires with no items completed (n=16), and of women with more than one child (n=195), 233 lactating women with one child were considered (see Table 1). Of them, 80 presented a story of pregnancy loss. Overall, the median age was 34 years (IQR 31-37), and most women had a university degree (n=141; 60.52%), with no significant difference among women with versus without history of pregnancy loss.

The median age of newborns was 12 months (5-19). With regard to the type of delivery, 112 and 41 women (48.07 and 17.60%) had vaginal delivery without and with anesthesia, respectively, whereas 67 women (28.67%) had a C-section. Cesarean birth was significantly more frequent among women with history of pregnancy loss (n=33; 41.25%) as compared to control women (n=34; 22.22%) (*p=0.004*).

Information on breastfeeding is reported in Table 2. Out of 233 women, 184 (78.97%) declared that they have received information on the importance of breastfeeding. Exclusive breastfeeding was reported by 196 women (84.12%), and median duration of exclusive breastfeeding was 6 months (4-7). Considering difficulties experienced during breastfeeding, a significant proportion of women reported breast fissures (n=77; 33.05%), engorgement (n=76; 32.62%), and poor milk production (n=29; 12.45%). Proportion and length of exclusive breastfeeding, as well as occurrence of specific complications related to breastfeeding, were comparable among women with versus without history of pregnancy loss.

Use of CAMs during breastfeeding and during pregnancy among women with or without history of pregnancy loss is described in Table 3. In both cohorts, a notable proportion of women reported use of CAMs during pregnancy (n=70; 45.75% versus n=35; 43.75% among control women versus women with history of pregnancy loss, respectively;* p=0.759*). Furthermore, more than half of interviewed women declared use of CAMs during ongoing breastfeeding: in particular, use of CAMs in breastfeeding was more frequent among women with history of pregnancy loss (n=82; 53.59% versus n=54; 67.50% among control women versus women with history of pregnancy loss, respectively;* p=0.050)*. Specifically, women with history of pregnancy loss more frequently resorted to herbal preparations (n=25; 16.34% versus n=24; 30.00% among women without versus with history of pregnancy loss;* p=0.018*), whereas phytotherapics were among the most frequently taken CAMs in both cohorts (n=33; 21.57% and n=21; 26.25%, respectively). Overall, CAMs products were most frequently reported to contain galactagogues or multivitamin and mineral supplements.

Advise for CAMs use was mainly provided by midwives, gynecologists, and friends or family members, although in both groups a relevant proportion of women resorted to CAMs on a self-prescription basis (n=17; 11.11% and n=13; 16.25% among control women and women with history of pregnancy loss, respectively;* p=0.305*).

**Figure 1:**
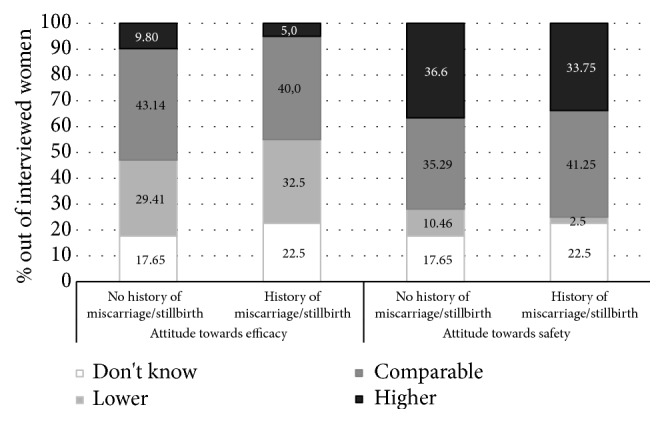
Attitude of interviewed women towards CAMs efficacy and safety. *∗*p=0.121 and p=0.500 for efficacy and safety.

The attitude of women towards efficacy and safety of CAMs is described in Figure 1. Regarding efficacy, only 9.80% and 5.00% of women without and with history of pregnancy loss believed that CAMs had higher efficacy than traditional medications, 43.14% and 40.00% believed that CAMs were as effective as traditional medications, and 29.41% and 32.50% considered CAMs' efficacy as lower. Perception on efficacy was comparable among women without and with history of pregnancy loss (*p=0.121*). 36.60% and 33.75% of women without and with history of miscarriage or stillbirths were convinced that CAMs were more safe than conventional medications, whereas 35.29% and 41.25% believed that CAMs were as safe as traditional medications, and 10.46% and 2.50% considered CAMs less safe. Perception on safety was comparable among women without and with history of pregnancy loss (*p=0.500*). Of note, 17.65% and 22.50% of women without and with history of pregnancy loss reported that they have no clear perception of the efficacy and safety of CAMs.

**Figure 2:**
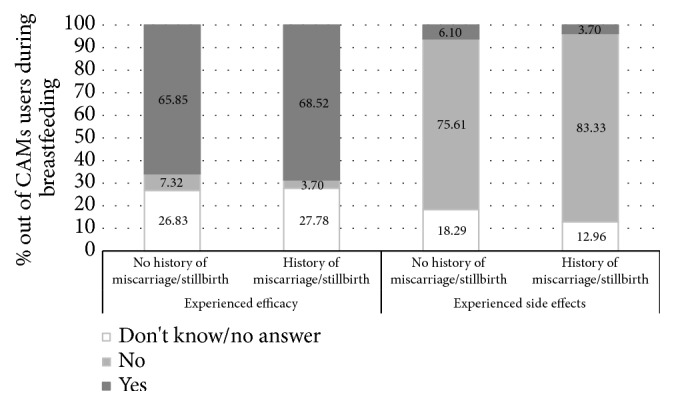
Benefits and side effects reported by CAMs users during breastfeeding. **∗**p=0.762 and p=0.593 for benefits and side effects.

Real benefits and side effects reported by the 82 and 54 women without and with history of pregnancy loss who used CAMs during breastfeeding are reported in Figure 2. Most women (65.85 and 68.52% of women without and with history of pregnancy loss) reported benefits related to the use of these products. Notably, benefits most frequently reported were related to breast fissures (n=32), engorgement (n=34), and poor milk production (n=12).

Regarding safety, only 7 women (5 control women and 2 women with history of pregnancy loss) declared that they have experienced side effects during CADs use. In particular, one woman reported mental confusion, one reported fetal tachycardia, and one stomachache, whereas a total of four women did not specify the experienced side effect.

## 4. Discussion

To the best of our knowledge, this is the first Italian study characterizing the attitudes towards the use of CAMs among Italian breastfeeding women with history of pregnancy loss. Results from this* post hoc* analysis in the framework of the HaBIT study [15] confirm that the use of CAMs during breastfeeding is widespread among Italian women at first breastfeed. Most frequently used CAMs were herbal preparations, followed by phytotherapics and dietary supplements. Specifically, results show that use of CAMs is significantly more frequent among women with previous history of pregnancy loss, as compared to control mothers. This finding is of particular relevance, considering that both interviewed groups reported comparable frequencies of lactation-related complications, mainly represented by breast fissures, engorgement, and poor milk production, over a comparable median duration of exclusive breastfeeding.

Thus, more frequent use of CAMs among women with history of pregnancy loss could be related to psychological rather than clinical issues, probably ascribable to adverse pregnancy-related outcomes. It is increasingly recognized that the negative psychological and mental health consequences related to pregnancy loss continue also in subsequent pregnancies [23, 24]. Literature evidence reports higher levels of psychological distress, pregnancy-specific anxiety, and depression among pregnant women with history of loss [25, 26]. In this context, it is likely that interviewed women with previous adverse pregnancy more frequently resorted to CAMs to face consequences related to the previous pregnancy loss. Rationale for higher use of CAMs among women with history of loss may also rely on the higher frequency of cesarean delivery in this group as compared to controls, with women resorting to CAMs due to complications related to this surgical procedure (i.e., postsurgical pain). Independently from its possible association with CAMs use, our finding reporting significantly higher rates of cesarean delivery among women with history of pregnancy loss is of particular interest. It is well described in literature that pregnancies subsequent to losses are characterized by increased rates of interventions such as induction of labor and elective cesarean delivery, even when they are not associated with an increased risk of subsequent loss [12]. A study on the management of pregnancy after unexplained stillbirth revealed that elective cesarean section was often advised by midwives, irrespectively from other clinical considerations [27]; however, according to a web-based survey, 81% of women who experienced stillbirth wanted early delivery and 26% wanted a cesarean delivery in the subsequent pregnancy, independently of the medical and obstetrical advice [13, 28]. On the one hand, these mothers' wishes for the management of new pregnancies further highlight the anxiety and the psychological burden related to previous loss. On the other hand, these considerations further emphasize the need for an accurate clinical plan for the management of pregnancy, delivery, and postnatal care following losses, in order to provide the most appropriate psychological and obstetrical assistance, while minimizing the risk of unnecessary interventions.

The importance of building a solid woman-midwife relationship, founded on a specific professional expertise as well as on a human and medical trust, is acknowledged also by our finding; in fact, midwives were the major advisors for CAMs use during breastfeeding, although a nonnegligible proportion of CAMs use was based on self-prescription or familiar advice. Despite being advised about CAMs use by midwives, a significant proportion of CAMs users reported that they have no precise information regarding CAMs use during breastfeeding. Nevertheless, a proportion (although minor) of interviewed CAMs users actually reported that they have experienced adverse events related to these products.

There is growing interest in the use of Internet-based surveys for medical research, particularly when investigating on sensitive topics. In fact, while accounting for several advantages such as electronic dexterity, reduced time from research question to answers, reduced error rates in data collection, and reduced research costs [29], the use of nonfacial survey for sensitive questions helps sharing information and experiences without inhibition [28]. Nevertheless, given the distance from interviewed subjects, the basic demographics of the respondents as well as true understanding of the questions could not be verified. Second, as for all surveys, provided answers could be affected by* recall bias*. Third, with this survey it was not possible to investigate details of the previous loss, particularly in terms of gestational week, and causes (i.e., unexpected or related to fetal congenital abnormalities). Finally, the questionnaire did not provide data on comorbidities and concomitant use of conventional drugs; thus, we could not evaluate the association between these factors and CAMs use.

## 5. Conclusions

The new pregnancy and perinatal period following miscarriage or stillbirth are usually accompanied by anxiety and psychological difficulties, not only for the couple but also for the caregivers. Overcoming the social taboo of pregnancy loss and training healthcare professionals for a psychologically and clinically adequate management of the perinatal period and related complications, with a particular focus on breastfeeding, are essential for an effective and safe care of both mothers and newborns. Although our results reported a common use and advice on CAMs use during breastfeeding, it is important for both mothers and healthcare professionals to acknowledge that limited evidence supports their safety and efficacy during such critical period.

## Figures and Tables

**Table 1:** Sociodemographic characteristics of interviewed breastfeeding women.

	**Breastfeeding women, overall** **n=233 (**%**)**	**No History of miscarriages/stillbirths** **n=153 (**%**)**	**History of miscarriages/stillbirths** **n=80 (**%**)**	***p-value⁎***
**Median age** (IQR), years	34 (31-37)	34 (30-37)	34 (32-37)	*0.396*

**Educational level**				
Middle school certificate	13 (5.58)	8 (5.23)	5 (6.25)	*0.871*
High school certificate	79 (33.91)	51 (33.33)	28 (35.00)	
University degree	141 (60.52)	94 (61.44)	47 (58.75)	

**Median age of newborn** (IQR), months	12 (5-19)	11 (5-17)	13.50 (6 -21)	*0.073*

**Type of birth**				
Vaginal without anesthesia	112 (48.07)	81 (52.94)	31 (38.75)	*0.004∗*
Vaginal with anesthesia	41 (17.60)	26 (16.99)	15 (18.75)	
Vacuum-assisted vaginal delivery	13 (5.58)	12 (7.84)	1 (1.25)	
Caesarian	67 (28.76)	34 (22.22)	33 (41.25)	

*∗*from Fisher exact test

**Table 2:** Information on breastfeeding.

	**Breastfeeding women, overall** **n=233 (**%**)**	**No History of miscarriages/stillbirths** **n=153 (**%**)**	**History of miscarriages/stillbirths** **n=80 (**%**)**	***p-value***
**Information on breastfeeding importance**	184 (78.97)	120 (78.43)	64 (80.00)	*0.866*

**Exclusive breastfeeding**	196 (84.12)	132 (86.27)	64 (80.00)	*0.258*

**Median duration of exclusive breastfeeding** (IQR), months	6 (4-7)	6 (4-7)	6 (4-7)	*0.823*

**Supplements**				
Water	4 (1.72)	2 (1.31)	2 (2.50)	*0.609*
Artificial milk	34 (14.59)	20 (13.07)	14 (17.50)	*0.435*
Tisane	5 (2.15)	4 (2.61)	1 (1.25)	*0.663*

**Breastfeeding-related difficulties**				
Mastitis	17 (7.30)	13 (8.50)	4 (5.00)	*0.431*
Breast fissures	77 (33.05)	49 (32.03)	28 (35.00)	*0.662*
Engorgement	76 (32.62)	51 (33.33)	25 (31.25)	*0.771*
Inverted nipple	19 (8.15)	15 (9.80)	4 (5.00)	*0.313*
Poor milk production	29 (12.45)	18 (11.76)	11 (13.75)	*0.679*
Incompatibilities with work	10 (4.29)	7 (4.58)	3 (3.75)	*1.000*
Practical difficulties in breastfeeding management	27 (11.59)	19 (12.42)	8 (10.00)	*0.670*
Intolerance, discomfort	14 (6.01)	11 (7.19)	3 (3.75)	*0.391*
Difficulties with partner	6 (2.58)	3 (1.96)	3 (3.75)	*0.416*

**Table 3:** Use of CAMs during pregnancy and during breastfeeding.

	**No History of miscarriages/stillbirths** **n=153 (**%**)**	**History of miscarriages/stillbirths** **n=80 (**%**)**	***p-value***
**Use of CAMs in Pregnancy**			
No	81 (52.94)	45 (56.25)	*0.759*
Yes	70 (45.75)	35 (43.75)	
Missing	2 (1.31)	0	

**Use of CAMs in Breastfeeding**			
No	71 (46.41)	26 (32.50)	*0.050 (∗*)
Yes	82 (53.59)	54 (67.50)	

**Type of CAMs**			
Dietary supplements	26 (16.99)	17 (21.25)	*0.478*
Herbal preparations	25 (16.34)	24 (30.00)	*0.018∗*
Homeopathy	4 (2.61)	3 (3.75)	*0.694*
Phytotherapics	33 (21.57)	21 (26.25)	*0.419*
Traditional practices	7 (4.58)	1 (1.25)	*0.269*
Galenic preparations	1 (0.65)	0	*n.c.*
Domestic preparations	1 (0.65)	0	*n.c.*
Acupuncture	0	1 (1.25)	*n.c.*
Others	8 (5.23)	2 (2.50)	*0.501*

**Advise for CAMs use**			
Self-prescription	17 (11.11)	13 (16.25)	*0.305*
General practitioner	3 (1.96)	2 (2.50)	*1.000*
Gynaecologist	19 (12.42)	9 (11.25)	*1.000*
Paediatrician	17 (11.11)	8 (10.00)	*1.000*
Midwife	28 (18.30)	14 (17.50)	*1.000*
Pharmacist	5 (3.27)	3 (3.75)	*1.000*
Herbalist	8 (5.23)	10 (12.50)	*0.068*
Naturopath	4 (2.61)	2 (2.50)	*1.000*
Internet	11 (7.19)	4 (5.00)	*0.588*
Phytotherapy expert	8 (5.23)	5 (6.25)	*0.769*
Friends/family	17 (11.11)	13 (16.25)	*0.305*
Others	3 (1.96)	4 (5.00)	*0.236*

## Data Availability

The data used to support the findings of this study are available from the corresponding author upon request.
